# Some Observations on the Medicinal Effects of the Lichen Islandicus and Arnica Montana

**Published:** 1789

**Authors:** Alexander Crichton


### LONDON MEDICAL JOURNAL.

I. *Some Observations on the medicinal Effects of the Lichen Islandicus and Arnica Montana. Communicated in a Letter to* Samuel Foart Simmons, *M. D. F. R. S., by* Alexander Crichton, *M. D.*

I HAVE the honour of sending you enclosed some observations, written with a view to ascertain the medicinal qualities of the lichen islandicus and arnica montana, two remedies at present but little known to the medical practitioners of this island.

What principally induces me to trouble you with this account is, that, in a celebrated and justly-admired work on the materia medica, which has lately made its appearance, and in which one might naturally have expected an account of every thing new on the subject, no mention whatever is made of the first of these remedies; and for an account of the second, or arnica montana, the learned author refers his readers entirely to the account given of of it by Dr. Collin, of Vienna. But as, in my opinion, Professor Collin has endeavoured to raise too high expectations of this medicine, and has ascribed to it too extensive and general powers, I am desirous to prevent, as much as lays in my power, any disappointment which may be experienced in the use of it, by stating to you the opinions of other physicians concerning the arnica, and relating what I myself have had occasion to witness of its effects.

### *Of the Lichen Islandicus.*

When the lichen islandicus is boiled or infused in water, it yields a very thick mucilage, of a penetrating bitter and somewhat astringent taste; qualities which might induce one *a priori* to suppose it possessed of good medicinal qualities.

In dysentery, in certain species and periods of phthisis pulmonalis and hectic fever, and in that troublesome and obstinate cough which remains after the measles, this remedy has been greatly, and, in my opinion, deservedly extolled, not only by many of the German, but by some of the Swedish and French physicians. At the same time its use even in those countries seems to be far from being general; many looking looking upon it both as a very inefficacious and hurtful medicine. The truth is, the difeafes in which this remedy has been fo much recommended differ fo greatly, not only at different times, but in each individual from the peculiarities of conftitution, that fome difference in the treatment is abfolutely requifite; and medicines which are proper in one cafe, and in one ftage of thefe difeafes, are altogether improper in others. But, unhappily, moft of thofe who have written any thing about the virtues of the lichen iflandicus, if we except Dr. Marcus Herz, of Berlin, have been but little accurate in their accounts refpecting the fpecies of the difeafes in which this remedy has been found ufeful, or the time beft calculated for its adminiftration: hence we may eafily account for its inefficacy in the hands of fome, and its injurious effects in thofe of others.

In dyfentery there are certain circumftances which certainly forbid the ufe of the lichen iflandicus. Among the principal of thefe may be mentioned a fixed pain in any part of the abdomen, efpecially if the pulfe at the fame time is quick and hard, the fkin hot, and the patient thirfty; and indeed it ought never to be adminiftered where the phyfician fufpects any inflammatory affection; neither fhould it be exhibited in any cafe in which, from the appearance of the ftools, the uneafinefs of the patient, and the frequency of the tormina, it may be fuppofed that a quantity of hardened fæces remains in any part of the tract of the inteftinal canal.

The fum of the fymptoms which the patients laboured under, whom I either faw or heard of being cured by the lichen iflandicus, amounts to the following: — perfectly liquid, frequent, and more or lefs bloody ftools, attended with tenefmus and tormina, fometimes very violent and diftreffing; a quick, but not a hard pulfe, often foft and feeble; great and univerfal debility; and lofs of appetite.

I have feen patients labouring under the greateft complication of thefe fymptoms entirely cured by the lichen; and Dr. Herz, in his Letters addreffed to Phyficians *, enumerates a number of fimilar cafes alfo cured by it.

This celebrated phyfician, who gives an excellent and candid ftatement of the effects which he has experienced from it, accompanied

* Briefe an Aerzte.

with the hiſtory of ſome of the caſes, ſays, that, from the time when he firſt uſed this remedy in dyſentery, he has never had occaſion to have recourſe to any other. — " As ſoon," ſays the Doctor, " as I have ſufficiently cleanſed " the ſtomach and inteſtines, I betake myſelf " to the lichen iſlandicus; only that, according " to taſte, or other circumſtances, I ſometimes join a ſyrup to it, and now and then a " little opium."

In Vienna the lichen iſlandicus is held in high eſtimation in the phthiſis pulmonalis, a diſeaſe exceedingly frequent in that city. During a ſeven-months ſtay there, and a pretty conſtant attendance at the General Hoſpital, I had frequent opportunities of ſeeing this remedy tried for the cure of the diſeaſe alluded to. I am ſorry, however, to be obliged to confeſs, that it by no means anſwered the expectation I had formed of it.

From what I have ſeen, I am fully convinced, in my own mind, that there are only two ſpecies of this diſeaſe where this ſort of lichen promiſes a cure. The two ſpecies I hint at are, the *phthiſis hæmoptoica*, and the *phthiſis pituitoſa* or *mucoſa*. In ſeveral caſes of theſe I have ſeen the patients ſo far get the better of their complaints as to be dismissed the hospital cured; but whether they remained long so or not I cannot take upon me to say.

In both these species of phthisis the lichen islandicus is inadmissible, should there be a fixed pain in any part of the thorax, increased by a deep inspiration, and attended with a quick and hard pulse. The reason of this will be evident to any one who considers the cause of such symptoms, and the general effects of this lichen.

The cases of phthisis hæmoptoica, which I thought were cured by it, have not been very numerous. In some I have seen it fail, especially in such as seemed strongly predisposed to this disease by their original conformation: but where the rupture of the blood vessels of the lungs seemed to have arisen accidentally from external violence, from exertions, or from passion, and where the wound had run into suppuration from neglect, or an injudicious treatment, if the patient was not otherwise much predisposed to the disease, and his strength already not greatly exhausted, the lichen islandicus had always the happiest effect.

The good qualities of the lichen islandicus are more certain and constant in the phthisis pituitosa.

pituitofa, as it is called. I am afraid, however, that in this country we ſhall find leſs benefit from this medicine, in conſumptive caſes, than may by many be expected, as I am confident that, on an average, nine caſes out of ten of our conſumptions are owing to a ſcrophulous affection of the lungs. In this ſpecies, commonly diſtinguiſhed by the name of *phthiſis tuberculoſa*, I have often ſeen trials made with this remedy, and, I am ſorry to add, have as often ſeen it fail. Dr. Herz, of Berlin, whom I have already ſpoken of, takes alſo particular notice of this; but mentions the ſucceſs he has derived from it in the phthiſis hæmoptoica, and gives a hiſtory of ſome of the caſes to ſtrengthen his aſſertion. It is on the authority of this gentleman alone that I pretend to recommend the lichen to the attention of your readers in the cough which, now and then, remains ſo obſtinately, for a length of time, after the meaſles.

The lichen iſlandicus is commonly given in the form of a decoction; an ounce and a half of the lichen being boiled in a quart of milk. Of this a tea-cupful is directed to be drank frequently in the courſe of the day. If milk diſagrees with the patient's ſtomach, a ſimple

 decoction decoction of the lichen in water will anſwer very well. Care ought to be taken that it be boiled over a ſlow fire, and not longer than a quarter of an hour, as it is apt otherwiſe to loſe ſome of its qualities.

The lichen ſeems always to have one evident effect, that of ſtrengthening the powers of digeſtion, and indeed of the whole ſyſtem.

In dyſentery its peculiar evident effects ſeemed to be to alleviate the tormina, and that often very quickly; to diminiſh the frequency of the ſtools, and to change them to a more natural conſiſtence.

In phthiſis its good effects are pointed out by an amelioration of the matter expectorated; by diminiſhing the frequency of coughing, and rendering it alſo eaſier; by diminiſhing the irritability of the patient; and preventing or gradually moderating the hectic fever.

### *Of the Arnica Montana.*

The arnica montana has a penetrating bitter and ſomewhat aromatic taſte, which is ſtronger in the flowers than in the leaves, and weakeſt in the root.

Dr. Collin, of Vienna, in the years 1773 and 1775, endeavoured to recal this plant from oblivion oblivion by the publication of a number of cafes, and experiments made with a view to afcertain its qualities*. The refult of the cafes which he gives us tends to prove the arnica to be a very ufeful and efficacious remedy in putrid fevers, intermittents, palfies, tremors, and amaurofis.

It is, however, to be much lamented that Dr. Collin, from an over anxiety to ftamp a high character on the arnica, feldom informs us of any of the cafes but thofe in which it fucceeded.

In putrid fever I am happy to fay I can ftrengthen his affertions, at leaft as far as my word will have any credit. With Profeffor Stoll, whofe practice I had an opportunity of witneffing for four months previous to his death, the arnica was a favourite remedy in the difeafe alluded to; and not only with him, but with other phyficians of the General Hofpital of Vienna, I have feen it fucceed wonderfully well. Even in the worft ftages, where the pulfe has been exceedingly weak, fmall, and quick, where

* Vide Henr. Jofeph. Collin Obferv. circa morbos acutos et chronicos, Part. IV. Vienn. 1773, et ejufdem libri Part. V. ibid. 1775.

innumerable petechiæ have appeared, and even where the patients feemed exhaufted by a colliquative diarrhœa, this remedy generally produced the happieft effects. Dr. Stoll, however, gave it in much larger dofes than Dr. Collin recommends beginning with.

Effects fimilar to thofe juft mentioned I had again an opportunity of witneffing laft fummer, when attending the Clinical Hofpital at Gottingen. Profeffor Fifcher, who is phyfician to that hofpital, affured me that he had often experienced the happieft effects from it, both as an antifeptic and tonic remedy. I fhould be very forry were it to be underftood from what I have faid concerning this plant, that I am anxious to ftamp a higher value on it, as an antifeptic and tonic remedy, than on any other in common practice. The cafes in which I have feen it tried are not numerous enough to authorife my doing fo were I inclined. All I can fay is, that in this difeafe I do not recollect to have feen any other medicine neceffary when the arnica was freely and properly exhibited.

As to its effects in intermittents, I cannot fpeak from my own experience: but as it may afford fome fatisfaction to your readers to know the refult of the trials made with it in this difeafe, case, I shall take the liberty of transcribing here such of my notes as relate either to what I have learnt from the conversation of foreign physicians on this subject, or to what I have extracted from their writings.

Dr. Collin, in the fifth part of his *Observationes* &c. says he has cured thirty-eight quotidians, forty-six tertians, and fifty-eight quartans, with the arnica. His dose of the extract was about a drachm per diem.

Dr. Sebold, Professor of the Clinical Institution at Prague, assured me, whilst there in September, 1787, that, in the cure of intermittents, after cleansing the primæ viæ, he trusted entirely to the arnica and flowers of chamomile, and that in the many cases which had occurred to him of this disease this method had never disappointed his expectations.

In Jutland this plant is a common domestic remedy in the ague, and held in great esteem*. Dr. Manger says he has frequently experienced the best effects from an infusion of half an handful of the flowers drank two hours before the access of the paroxysm. All this tends, in my

* Vide Gesner's Entdeckungen in der Arzneiwissenschaft, 3 B. 2 theil.

opinion, to prove the tonic qualities of the arnica.

It has been much recommended in palſies and tremors; but I only remember to have ſeen one caſe of each of theſe diſeaſes got the better of under the adminiſtration of the arnica; and even in theſe caſes the arnica, perhaps, had not the ſole merit of the cure: for in the one it was joined to the uſe of valerian, and in the other to that of camphor. In two or three other ſimilar caſes which I ſaw treated with the arnica, during my ſtay at Vienna, it failed.

Dr. Collin ſays he has cured nine caſes of amauroſis with it.

Dr. Richter, the celebrated profeſſor of ſurgery at Gottingen, who has a great deal of practice in diſeaſes of the eyes, ſays, that he has tried the arnica very often in this diſeaſe, but that it did not anſwer his purpoſe ſo well as it ſeems to have done Profeſſor Collin's, although he confeſſes it to have been now and then of ſervice *.

Having now ſtated to you, as conciſely as I poſſibly could, ſuch information as I think may

* Richter's Chirurg. Bibliotheck, 2 Band, Gottingen, 1772.

throw throw ſome light on the medicinal qualities of the arnica, I ſhall only add a few words more on the different methods of exhibiting it, and on its apparent effects.

Either the whole plant may be uſed, or its flowers, or roots, in the form of a powder, decoction, infuſion, or extract.

The whole plant is generally uſed in infuſion or decoction, in the proportion of an ounce of it to a pound and a half of water; which quantity may be given, in doſes of a cupful, in the courſe of the day.

Of the flowers two or three drachms daily will, at firſt, in moſt caſes, be ſufficient; although I have ſeen near an ounce of them taken in the ſpace of twenty-four hours.

The extract made from the whole plant, which, in my opinion, is by far the moſt elegant and commodious way of exhibiting it, may be given at firſt to the extent of a drachm *per diem*.

The arnica, eſpecially its flowers, produces often very diſagreeable and uneaſy ſenſations to the patient. The chief and moſt common of theſe are, a ſharp pricking ſenſation over the whole ſurface of the body, an uneaſy ſenſation at the region of the ſtomach, ſometimes accom-

 panied panied with cardialgia, and with ſlight convulſive ſhocks, reſembling thoſe produced by electricity.

Another very curious and pretty conſtant effect of the arnica is that of its indicating the place where any injury has taken place, from an external cauſe, by augmenting the pain in the part, ſhould any exiſt; or by renewing it, ſhould it have only lately quitted the patient.

It may be remarked, that the root ſeldom produces any of theſe diſagreeable ſymptoms; whereas the flowers of the arnica ſeldom fail to do ſo. When theſe ſymptoms do take place, it is always looked upon as a ſure ſign of the remedy's taking a proper effect; therefore its uſe ought not to be diſcontinued on that account. A little of the extract of gentian generally prevents its occaſioning the uneaſineſs at the ſtomach whilſt it co-operates in its effects.

The arnica almoſt uniformly increaſes the ſtrength, but not the velocity, of the pulſe.

*Oxford Court, Cannon Street,*

June 23, 1789.

